# Supplementary material for: SARS-CoV-2 Variant of Concern 202012/01 Has about Twofold Replicative Advantage and Acquires Concerning Mutations
Source: Viruses. 2021 Mar 1;13(3):392. doi: 10.3390/v13030392 (PMC8000749; doi:10.3390/v13030392)
Supplement: Supplementary file 1 [file viruses-13-00392-s001.zip › viruses-1091769--Grabowski_et_al_SuppFig1.pdf]

viruses

Supplementary Figure 1

SARS-CoV-2 Variant of Concern 202012/01 has about twofold replicative advantage and is acquiring concerning mutations

Frederic Grabowski [1], Grzegorz Preibisch [2], Stanisław Giziński [3], Marek Kochańczyk [1,*] and Tomasz Lipniacki [1,*]

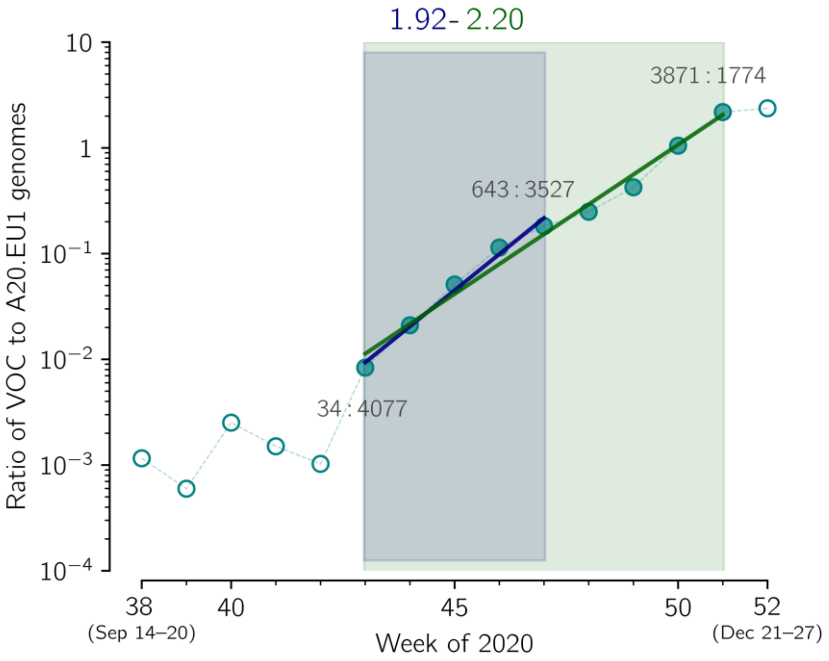

Figure S1. The replicative advantage of VOC over 20A.EU1 in England, based on COG. Ratio of VOC to 20A.EU1 genomes collected from pillar 2 tests in weeks 38-51 in England. The trend line is fitted to data points from weeks 43–51 (green) and from weeks 43–47 (blue). The weekly growth rate is 1.92 [95% CI: 1.80–2.04] for weeks 43–51 and 2.20 [95% CI: 1.98–2.43] for weeks 43-47. The estimated R ratio is Rt^{VOC} / $Rt^{20A.EU1}$ = 1.87 [95% CI: 1.76-1.98] for weeks 43-51 and 2.13 [95% CI: 1.93-2.35] for weeks 43-47.
